# Smartphone-assisted HPTLC for simultaneous determination of vonoprazan fumarate and aspirin: a comparative study with HPTLC densitometry

**DOI:** 10.1038/s41598-025-26418-x

**Published:** 2025-11-24

**Authors:** Sherin F. Hammad, Mohamed A. Adel Hamid, Samar H. Elagamy, Latifa Adly

**Affiliations:** 1 Department of Pharmaceutical Analytical Chemistry, Faculty of Pharmacy, Tanta University, Tanta, Egypt; 2 Department of pharmaceutical chemistry, Alsalam university, Tanta, Egypt

**Keywords:** Chemistry, Medical research

## Abstract

Smartphone-assisted HPTLC/Image J and HPTLC densitometric methods were developed and validated for the simultaneous determination of vonoprazan fumarate VON and aspirin ASP. Chromatographic separation was carried out using a mobile phase composed of methylene chloride, methanol, and glacial acetic acid in a 60:40:2.0 v/v ratio, yielding well-resolved and symmetric peaks with retention factor Rf values of 0.45 for VON and 0.75 for ASP. Densitometric analysis of chromatograms was performed at 270 nm, showing linearity in the range of 2.0–10 µg/band for VON and 5.0–25 µg/band for ASP. For the smartphone-assisted approach, captured plate images were processed using Image J software to quantify band intensities, providing linearity over 1.0–10 µg/band for VON and 5.0–35 µg/band for ASP. Both methods were validated according to ICH guidelines and successfully applied to pharmaceutical dosage forms, demonstrating good accuracy and precision. Statistical comparison with a reported HPLC method confirmed the reliability of the proposed approaches. Notably, the smartphone-assisted HPTLC/Image J method offers a cost-effective, accessible, and sustainable alternative to conventional HPTLC densitometry, while maintaining comparable accuracy and precision, making it a promising tool for routine pharmaceutical analysis.

**Supplementary Information:**

The online version contains supplementary material available at 10.1038/s41598-025-26418-x.

## Introduction

Vonoprazan fumarate VON is 1-[5-(2-fluorophenyl)−1-pyridin-3-ylsulfonylpyrrol-3-yl]-N-methylmethanamine fumarate Figure S1. It is a potassium-competitive acid blocker that competitively inhibits the potassium binding site of gastric H+/K + ATPase, and thus providing a promising alternative to proton-pump inhibitors PPIs^1^. VON is utilized for the treatment of acid-related disorders and as an adjunct in Helicobacter pylori (H. pylori) therapy^2^. Few analytical methods have been reported for the determination of VON in dosage form including chromatographic^3,4^ and fluorimetric methods^5^. A sensitive fluorimetric method has also been described for its quantification in human plasma^6^. In addition, a chromatographic method has been developed for the simultaneous determination of VON in combination with other drugs such as metronidazole and amoxicillin^7^. Another chromatographic method has also been reported for its determination in binary and ternary mixtures with metronidazole, amoxicillin, clarithromycin, and aspirin^8^.

Aspirin ASP, 2-acetoxybenzoic acid, is an analgesic and antipyretic medication Figure S2. Its mechanism of action involves inhibiting cyclooxygenase COX^9^, which reduces the formation of prostaglandins PGs responsible for inflammation, swelling, pain, and fever^10^. It also has an antiplatelet effect due to the inhibition of platelet COX, necessary for synthesizing thromboxane in platelets, thereby reducing thromboxane levels that facilitate platelet aggregation and activation. Several chromatographic methods have been reported for the determination of aspirin, either in the presence of its degradation products or in combination with other drugs^11,12^. An electrochemical method has also been developed for the simultaneous determination of aspirin and caffeine in human urine samples^13^. Additionally, various spectrophotometric methods have been applied for the determination of aspirin in combination with other analgesics in pharmaceutical dosage forms^14–16^.

Cabpirin^®^ tablets are approved in Japan for managing acid-related diseases in patients who need low-dose ASP but are at risk of ASP-induced gastric ulcers. This medication contains a combination low-dose ASP 100 mg and VON 10 mg. It is indicated for patients with a history of gastric ulcer or duodenal ulcer. To reduce the risk of thrombosis or embolism associated with conditions such as angina, myocardial infarction, cerebral infarction, or after operations such as coronary artery bypass graft (CABG). Only a few analytical methods have been reported for the simultaneous determination of VON and ASP. A spectrophotometric method has been described based on the ratio difference RD and first derivative of ratio spectra 1DD approaches^17^. Chromatographic methods have also been applied, including HPLC methods^8,18^ and a conventional HPTLC method^18^. However, no study to date has explored the use of smartphone-assisted HPTLC for this drug combination.

Image J is an open-source image processing software developed by the United States National Institutes of Health (NIH) for quantitative analysis of scientific images^19^. Recently, Image J has found wide applications in pharmaceutical analysis^20–22^ and environmental analysis^23,24^. Compared to various commercial platforms, Image J offers several advantages: it is freely available, user-friendly for basic analytical tasks without requiring prior expertise, and compatible across multiple operating systems such as Windows, macOS, and Linux^25^.

This study aims to develop and validate both smartphone-assisted HPTLC and conventional HPTLC densitometric methods for the simultaneous determination of VON and ASP in pharmaceutical formulations. The work further explores the use of Image J software for quantitative analysis of chromatographic bands from smartphone-captured images, offering a novel, eco-friendly, and cost-effective alternative to traditional densitometric detection. This approach enables HPTLC analysis using TLC sheets and a UV lamp, without the need for sophisticated equipment, thereby making the analysis more accessible and cost-effective.

## Experimental

### Apparatus

The spots were visualized using a UV lamp with a short wavelength of 254 nm (SPECTROLINER Model ENF-260 C, U.S.A.). Chromatographic separation was performed on TLC aluminum plates (20 × 20 cm, 0.2 mm) pre-coated with silica gel 60 F254 purchased from Merck (Darmstadt, Germany). Samples were applied using a CAMAG Linomat V automatic sample applicator (Muttenz, Switzerland) with a 100-µL microsyringe. Densitometric scanning of the obtained spots was carried out using a CAMAG TLC Scanner 3 (Camag, Switzerland), operating in absorbance mode and controlled by WinCATS software, version 1.4.1 (Camag, Switzerland). The scanning speed was 20 mm/s with a slit dimension of 6.0 mm × 0.3 mm, and a data resolution of 100 nm/step. Densitometric analysis of chromatograms was performed at 270 nm (± 1 nm).

### Materials

VON (100.54%) and ASP (100.23%) were kindly supplied by El Andalos Company (Egypt). Methylene chloride, methanol, and glacial acetic acid were purchased from EL-NASR Pharmaceutical Chemicals (Egypt).

### Standard solutions

Stock solutions of VON were prepared by dissolving 25 mg of the drug in 25 mL of methanol, resulting in a solution with a concentration of 1000 µg/ml. Working solutions were then prepared by diluting 1–10 mL of the stock solution when necessary to obtain a series of concentrations ranging from 100 to 1000 µg/mL. Whereas the stock solutions of ASP were prepared by dissolving 125 mg of the drug in 25 mL of methanol, yielding a solution with a concentration of 5000 µg/ml. Working solutions were then prepared by diluting 1–10 mL of the stock solution when necessary to obtain a series of concentrations ranging from 500 to 5000 µg/mL.

### Chromatographic conditions

Samples were applied as bands on TLC aluminum plates (20 × 10 cm, 0.20 mm) pre-coated with silica gel 60 F254. The band width was set to 6 mm, with each band spaced 1 cm apart and positioned 1 cm from the bottom edge of the plate. The chromatographic chamber was pre-saturated with the mobile phase for 30 min. Development was performed by the ascending technique with a mobile phase composed of Methylene Chloride: Methanol: Glacial Acetic Acid (60:40:2, by volume), up to a distance of approximately 8 cm. After development, the plates were air-dried at room temperature and scanned at 270 nm using the CAMAG TLC Scanner 3, operated in absorbance mode with a deuterium lamp as the radiation source.

### Construction of calibration curves

Aliquots of 10 µL from working standard solutions (100–1000 µg/mL for VON and 500–5000 µg/mL for ASP) were applied to TLC plates to achieve concentrations ranging from 1.0 to 10 µg per band for VON and 5.0–50 µg per band for ASP. Calibration curves were constructed by plotting the peak areas of the drugs at 270 nm against their concentrations. The resulting regression equations were used to determine the concentration of each drug. Images of the TLC plates were captured using a mobile camera, and the peak areas under the bands were quantified using Image J software following the procedure described in Appendix A of the Supplementary Information. Calibration curves were then constructed by plotting these peak areas against the corresponding concentrations.

### Assay of dosage form

Ten tablets, each with label claims of 10 mg VON and 100 mg ASP, were ground and thoroughly mixed. Powder equivalent to one tablet was dissolved in 50 mL of methanol, sonicated for 15 min, and then filtered. A 5.0 mL aliquot of this solution was transferred to a 10 mL volumetric flask and diluted to the mark with methanol, achieving final concentrations of 100 µg/mL for VON and 1000 µg/mL for ASP, which was used for the assay. Aliquots (10 µL) from this solution were applied to TLC plates, resulting in concentrations of 1.0 µg per band for VON and 10 µg per band for ASP.

## Results and discussion

### Optimization of chromatographic conditions

**Fig. 1:**
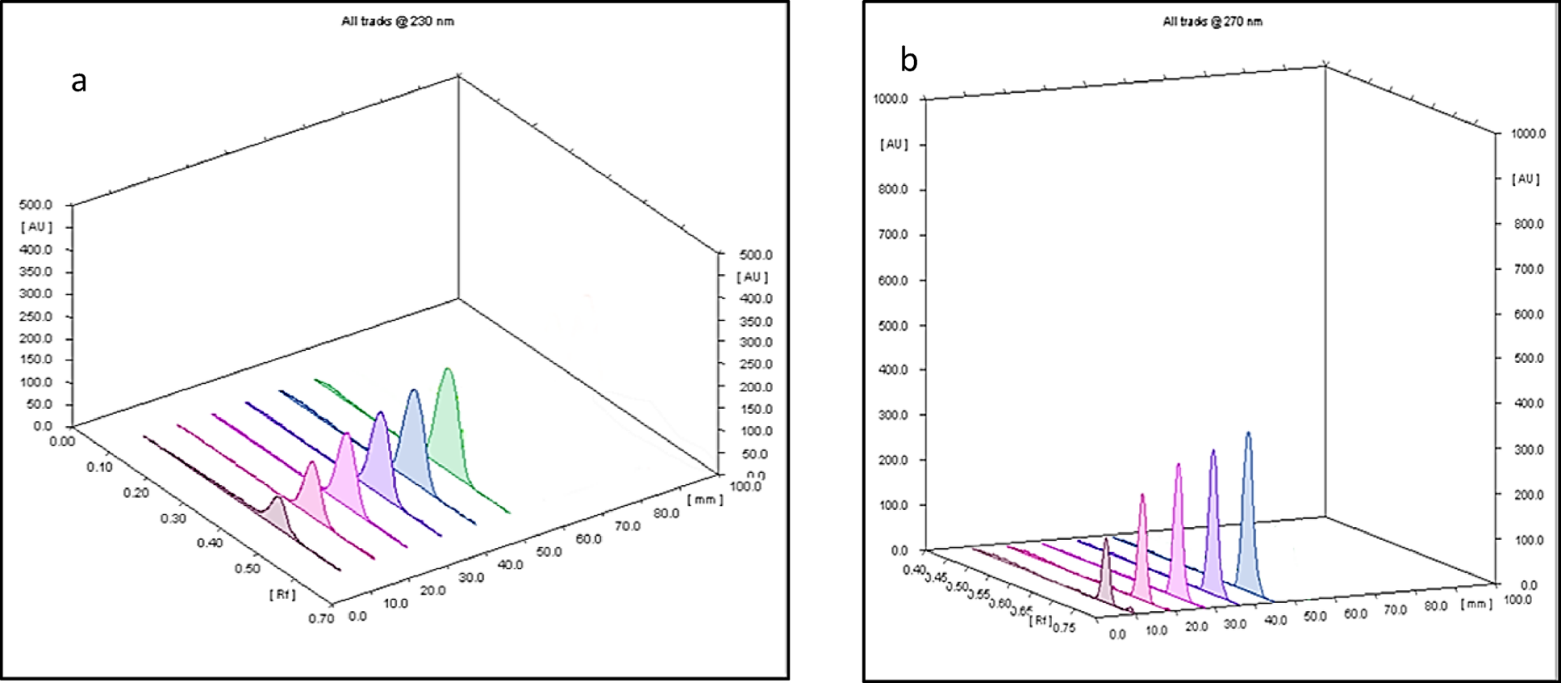
3D thin layer chromatography-densitogram of VON and ASP measured at 230 nm and 270 nm, respectively, for (**a**) different concentrations of VON(2.0–10 µg/band) and (**b**) ASP (5.0–25 µg/band).

**Fig. 2:**
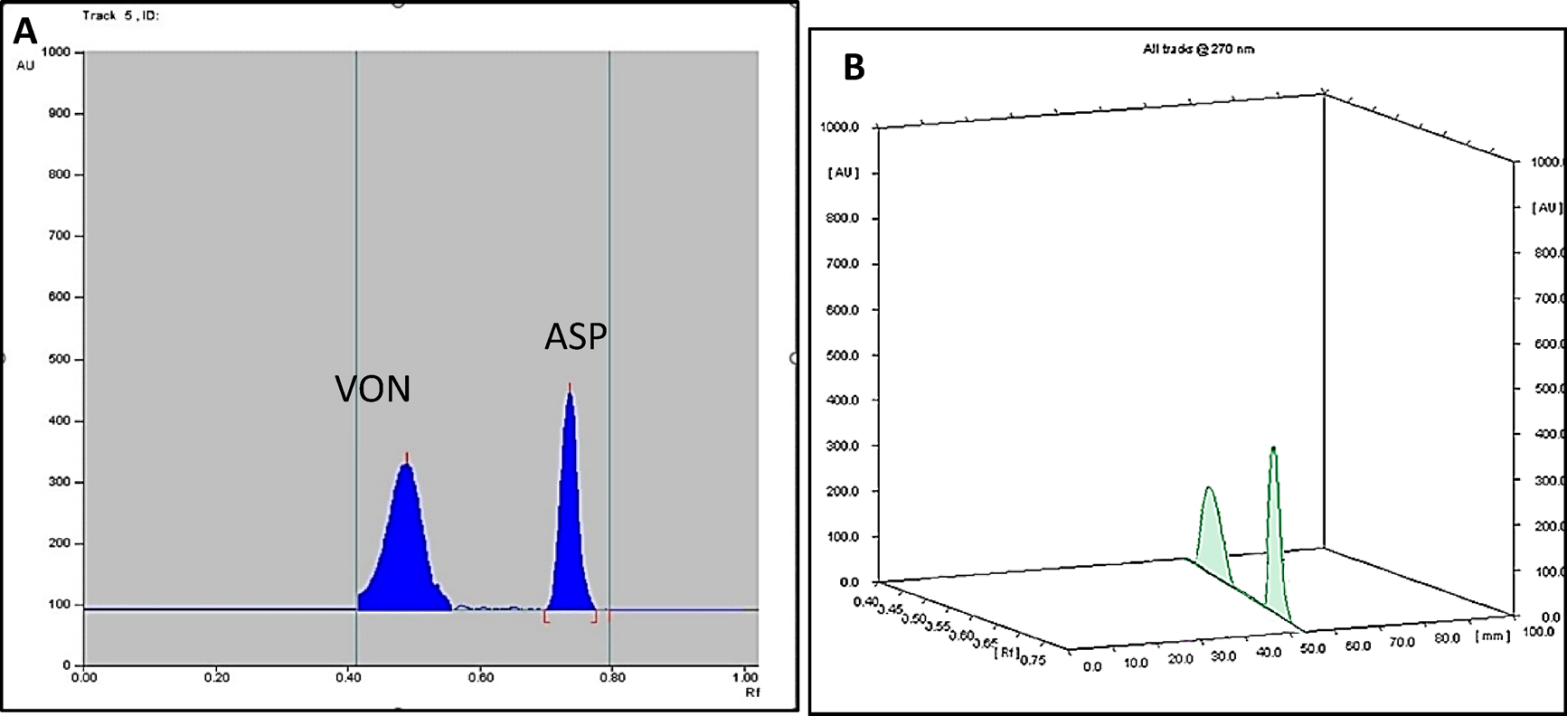
2and 3Dimensions thin layer chromatography-densitogram (**A** and **B**, respectively) for a binary mixture containing 1.0 µg/band VON, and 15 µg/band ASP.

The chromatographic conditions were optimized to achieve better resolution through a trial and error approach. Various mobile phases with different compositions and solvent ratios, ranging in polarity, were tested to obtain satisfactory separation. Initially, heptane and ethyl acetate in different ratios were evaluated, but both drugs remained at the baseline. Subsequently, a mixture of methylene chloride and methanol was tested, with a 60:40 ratio providing the best resolution. To further enhance spot shape and achieve well-defined concentric spots, glacial acetic acid was added. The scanning wavelength was carefully optimized to achieve the best results. Optimal scanning wavelength was determined by assessing various wavelengths according to the UV spectrum of both drugs Figure S3. A scanning wavelength of 270 nm was chosen as optimal wavelength, providing excellent sensitivity and satisfactory resolution. The resulting 2D and 3D TLC-densitograms for VON and ASP, as well as their binary mixture under optimal chromatographic conditions, are illustrated in Figs. 1 and 2.

### Application of image J for peak area measurement

**Fig. 3:**
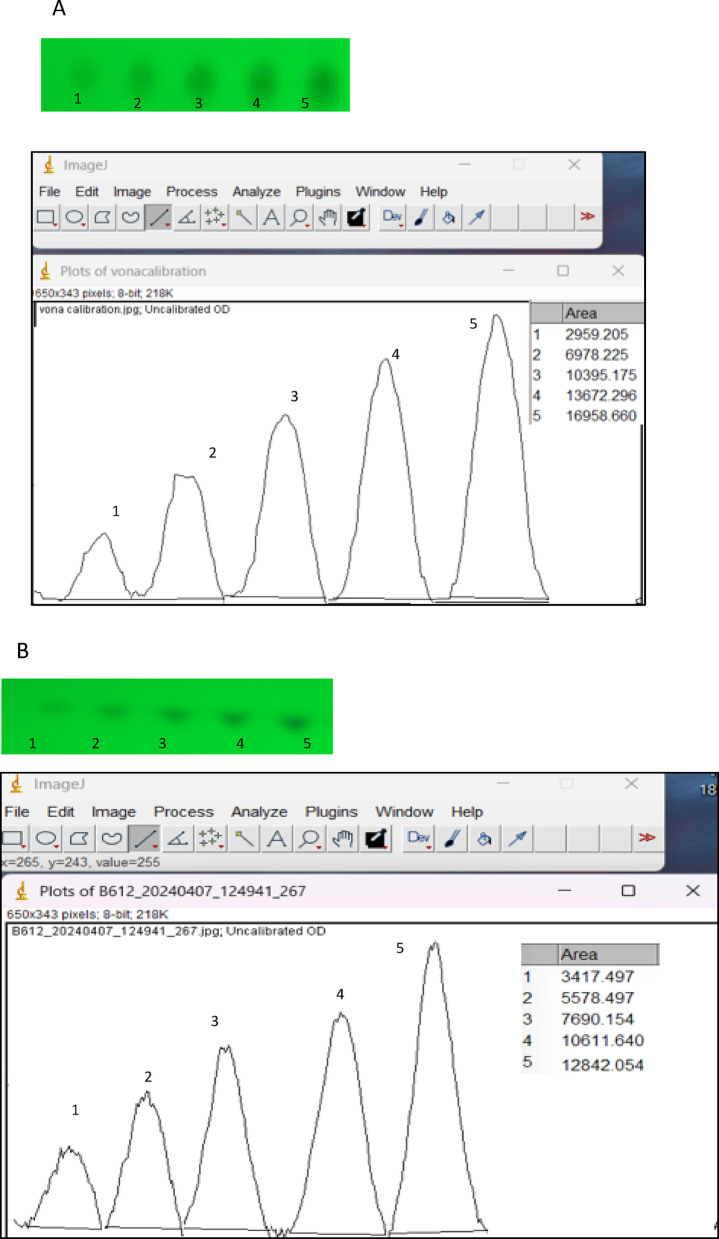
(**A**) Peak area measurement using Image J for successive concentration of VON (2.0, 4,0, 6.0, 8.0, 10 µg/band), (**B**) Peak area measurement using Image J for successive concentration of ASP (5.0, 10, 15, 20, 25 µg/band).

Image J offers the capability to measure peak areas under TLC bands. Instead of using TLC densitometry, spots were visualized under a UV lamp, and images were captured using a mobile phone camera. These images were then analyzed with Image J to determine the peak area of each spot. Figure 3 illustrates how Image J generates a series of line graphs that relate to the color density in each band. The original TLC silica gel plates are presented in Figure S4 and the steps for using Image J to measure peak areas are detailed in Appendix A in supplementary information.

### System suitability parameters

System suitability testing was conducted to evaluate the performance of the chromatographic system according to USP guidelines^26^. Parameters such as retardation factor (Rf), resolution (Rs), tailing factor (T), capacity factor (K’), and selectivity factor (α) were assessed. The chromatographic peaks for VON and ASP displayed symmetric profiles without tailing, as indicated by the tailing factor (T) values. The selectivity factor values for VON and ASP peaks were within the acceptable range, confirming adequate separation of the drugs. The resolution between the two peaks was determined to be 1.96, indicating effective separation^27^. The results are summarized in Table 1.

**Table 1:** System suitability parameters of the developed thin layer chromatographic-densitometric method.

Parameters	VON	ASP	Acceptance criteria
Retardation factor (RF)	0.45 ± 0.02	0.75 ± 0.02	-
Tailing factor (T)	1.0	0.98	~ 1.0
Capacity factor (K˜)	1.04	2	1–10
Selectivity factor (α)	1.923		> 1.0
Resolution (Rs)	1.9606		> 1.5

### Method validation

Method validation for the smart phone based HPTLC and HPTLC densitometry was performed by ICH Q2 (R1) guidelines^28^. The validation encompassed parameters such as linearity, specificity, accuracy, repeatability, and intermediate precision.

#### Linearity

The linearity ranges for both methods were 1.0 to 10 µg/band for VON and 5.0 to 25 µg/band for ASP. Calibration curves were constructed by plotting integrated peak areas against the concentration µg/band of each drug, demonstrating satisfactory linearity for both drugs. The regression equations showed a high correlation coefficient, indicating a strong linear relationship between the response and the concentrations of the drugs, as shown in Table 2.

#### Limit of detection (LOD) and limit of quantitation (LOQ)

The sensitivity of the developed methods was evaluated by determining the LOD and LOQ. These were calculated using the formulas: LOD = 3.3 σ/S and LOQ = 10 σ/S, where σ represents the standard deviation of the intercept and S is the slope of the calibration curve. The LOD values obtained were 0.306 µg/band for VON and 0.887 µg/band for ASP using the HPTLC densitometry method, while the smartphone assisted method yielded LOD values of 0.297 µg/band for VON and 1.288 µg/band for ASP, confirming the sensitivity of the developed methods, as detailed in Table 2.

**Table 2:** Regression parameters for determination of VON and ASP by the two proposed methods.

Parameters	HPTLC		HPTLC/IMAGE J	
	VON	ASP	VON	ASP
Linearity µg/band	1.0–10	5.0–25	1.0–10	5.0–25
Slope	1840.09 ± 27.37	329.386 ± 5.31	923.007 ± 13.075	338.66 ± 6.30
Intercept	2320.40 ± 170.96	1102.07 ± 88.06	−328.758 ± 83.2881	−580.96 ± 132.15
R	0.9999	0.9999	0.9998	0.9999
LOD µg/band	0.306	0.887	0.297	1.288
LOQ µg/band	0.92	2.67	0.92	3.90

#### Accuracy

The accuracy of both methods was assessed by calculating the recovery percentages of each drug, determined from their respective regression equations derived from the calibration curves of the developed methods. The recovery ranged between 98.0% and 102.0%. The mean percentage recovery ± standard deviation SD was calculated to evaluate the accuracy for different concentrations of the proposed binary mixtures of VON and ASP, with triplicate determinations recorded in Tables S1, S2.

#### Precision

Precision was evaluated by analyzing binary mixtures of VON and ASP. Intraday precision (repeatability) was determined by analyzing standard solutions of both drugs at three different concentrations within the linearity range three times on the same day. Interday precision (intermediate precision) was assessed by analyzing three different concentrations within the linearity range over three different days, with each concentration analyzed three times. The percentage relative standard deviation RSD% was calculated, demonstrating that both intraday and interday precision were achieved with RSD% values being less than 2 units, as summarized in Tables S3, S4.

#### Specificity

**Fig. 4:**
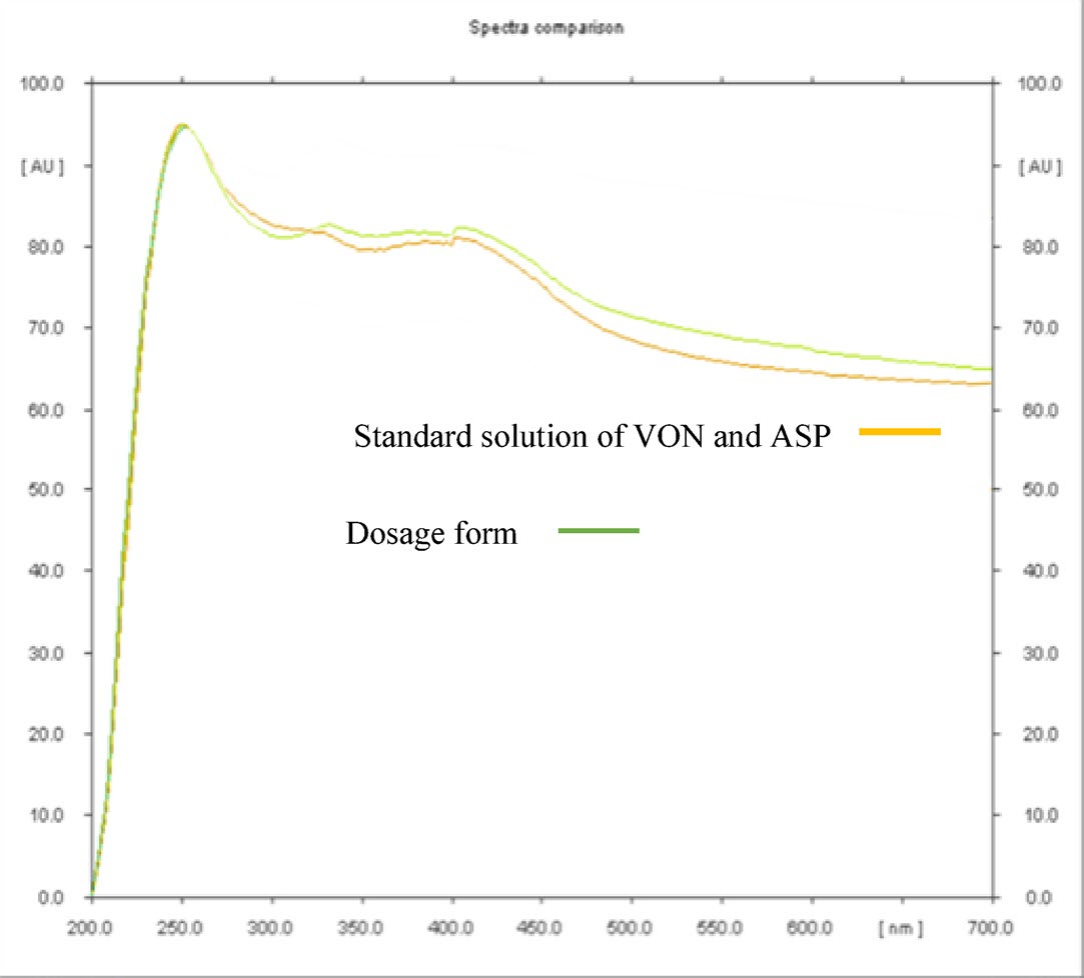
Peak purity curves of VON and ASP in a standard mixture and in the pharmaceutical dosage form.

The chromatogram of the dosage form displayed distinct and well-separated peaks for VON and ASP, and peak purity analysis confirmed the absence of interference from excipients Fig. 4. These results demonstrate that the proposed HPTLC densitometric method is valid and reliable for the analysis of VON and ASP in pharmaceutical formulations. The calculated percentage recovery values for the developed methods, when applied to the dosage form, further confirmed their specificity, with mean recoveries for both VON and ASP showing no significant interference from excipients Table 3. Moreover, statistical comparison using t-tests and F-tests, based on five determinations, indicated no significant difference between the results obtained by the developed methods and those reported for the previously published HPLC method^16^.

**Table 3:** Assay results for the determination of VON and ASP in Cabpirin^®^ tablets and statistical comparison to the methods.

Method	Drug	Mean % recovery* ±SD	t-value (2.228) ^a^	F-value (5.05) ^b^
HPTLC	VON	99.71 ± 0.777	0.105	2.02
	ASP	100.10 ± 0.95	0.347	1.41
HPTLC/Image J	VON	100.38 ± 0.59	2.51	1.36
	ASP	100.75 ± 0.76	1.94	1.56
The reported HPLC method^16^	VON	99.65 ± 1.105	………….	………….
	ASP	100.35 ± 1.31	………….	……………

^a, b^ are the theoretical t- value and F- value (0.05) at *n* = 6.

### The assessment of greenness, the blueness, and the whiteness of the proposed methods

As the HPTLC densitometric method offers several benefits, including higher accuracy and the ability to generate purity plots, the smartphone-assisted method provides practical advantages, such as improved accessibility, cost-effectiveness, minimal energy consumption, and suitability for resource-limited settings. Therefore, the sustainability and practicality of the two approaches were assessed using the Analytical Eco-Scale and the Blue Applicability Grade Index (BAGI), respectively. Analytical Eco-Scale assigns penalty points based on various factors, including the use and amount of hazardous chemicals, energy consumption, waste generation, and safety hazards^29,30^. The total penalty points are subtracted from 100 to determine the final Eco-Scale score, where a score of 75–100 indicates an excellent green method, 50–75 represents an acceptable green method, and values below 50 indicate inadequate greenness^31^. The smartphone-assisted HPTLC method achieved a score of 80, while the HPTLC densitometric method scored 79, confirming that both methods fall within the excellent green analysis category. The smartphone-assisted approach offers a slight advantage due to its lower energy consumption. The Eco-Scale assessment was also compared with previously reported HPTLC and HPLC methods, which achieved scores of 77 and 83, respectively, as detailed in Table S5. These results indicate that the developed methods provide better sustainability compared to the reported methods.

**Fig. 5:**
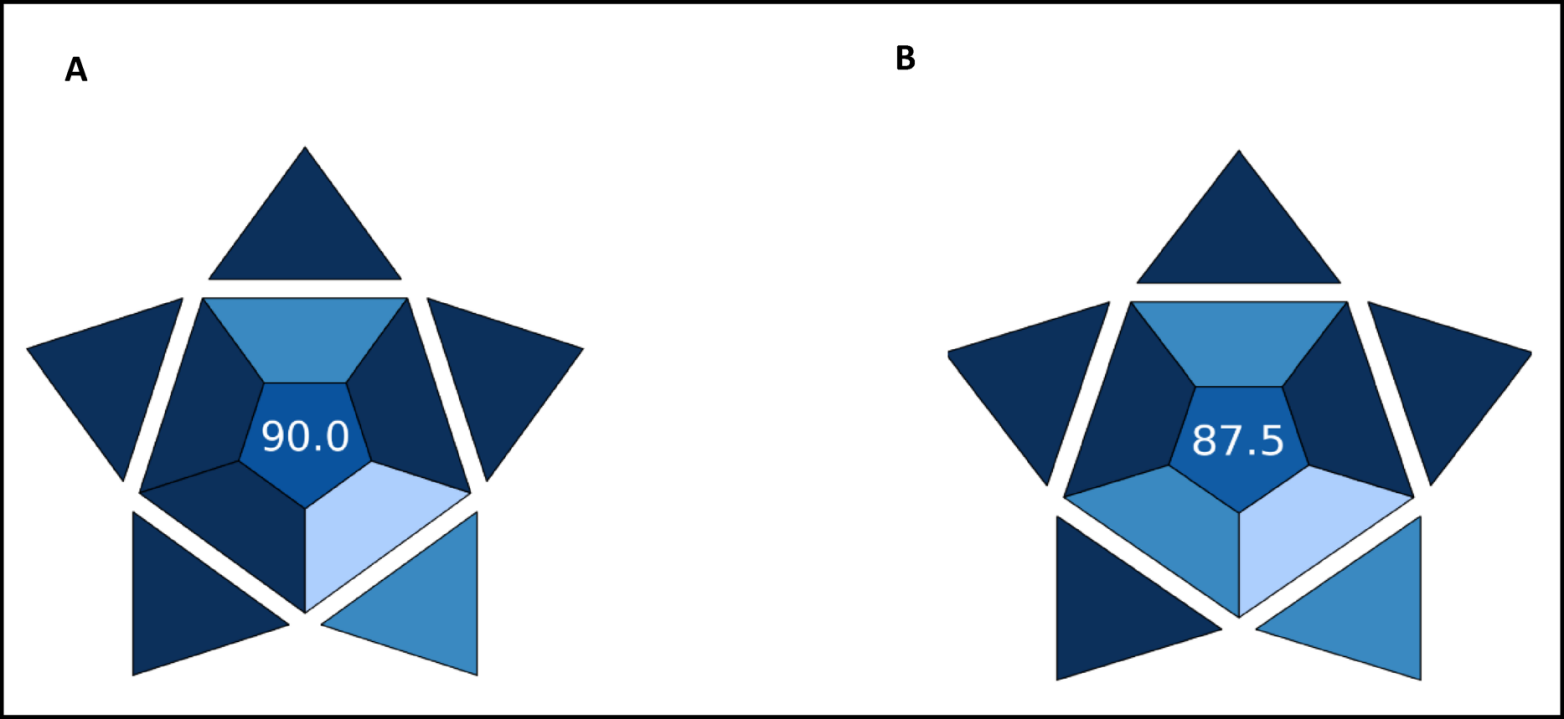
Results of BAGI evaluation of (**A**) HPTLC/Image J, (**B**) HPTLC densitometry.

As noted earlier, the smartphone-assisted approach is more practical than conventional HPLC. Therefore, the practicality of the developed methods was evaluated using BAGI. This index considers ten key attributes related to method applicability, including type of analysis, ability to quantify multiple analytes simultaneously, required techniques and instrumentation, sample throughput, preparation steps, reagents used, need for preconcentration, automation level, and sample size^32^. In this evaluation, the smartphone-assisted HPTLC approach achieved a BAGI score of 90, while the densitometric method scored 87.5, reflecting the smartphone-based approach’s enhanced accessibility and higher throughput Fig. 5.

**Fig. 6:**
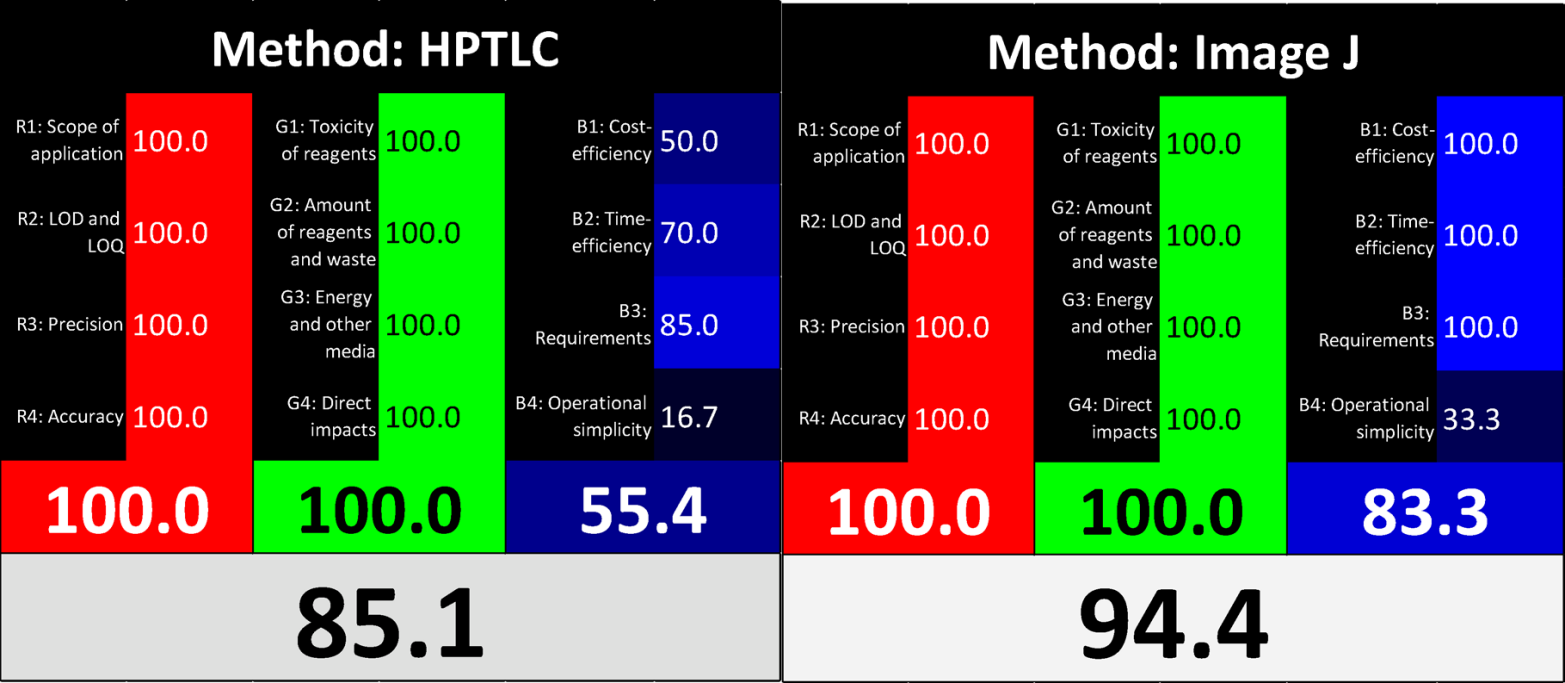
The whiteness evaluation of HPTLC densitometric method (Left) and smartphone assisted method (Right).

Finally, the whiteness of the two approaches was evaluated using RGB 12 model. The whiteness integrates three critical dimensions: analytical performance (red), environmental sustainability (green), and practical/economic efficiency (blue)^33–35^.As illustrated in Fig. 6, the smartphone-assisted HPTLC/Image J method achieved a Whiteness score of 94.4, compared to 85.1 for the HPTLC densitometric method. The higher score of the smartphone-based approach can be attributed to its portability, cost-effectiveness, and minimal energy consumption, highlighting its potential as a sustainable and widely applicable analytical tool.

### Comparison with other reported methods

The developed methods were compared with previously reported methods for the determination of VON and ASP in terms of linearity, sensitivity (LOD and LOQ), and sustainability, as summarized in Table 4. The developed methods demonstrated comparable sensitivity to reported chromatographic methods, but showed superior greenness, with the smartphone-assisted approach offering additional advantages of portability and accessibility.

**Table 4:** Comparison of the developed HPTLC densitometric and smartphone-assisted HPTLC methods with previously reported methods for the determination of VON and ASP.

Method	principle	Linearity µg/mL		LOD µg/mL		LOQ µg/mL		Ecoscale score	Refs.
		VON	ASP	VON	ASP	VON	ASP		
Spectrophotometric method	RD	1–10	2–25	0.173	0.24	0.525	0.728	86	^17^
	1DD	1–10	2–25	0.174	0.24	0.529	0.729		
Chromatographic	HPLC	0.5–10	1–100	0.17	0.33	0.5	1	82	^18^
	HPTLC	0.1–2 µg/band	0.1–10 µg/band	0.03 µg/band	0.03 µg/band	0.1 µg/band	0.1 µg/band	77	
Chromatographic	HPLC	1–100	1–100	0.24	0.29	0.80	0.97	70	^8^
Developed method	HPTLC	1–10 µg/band	5–25 µg/band	0.03 µg/band	0.03 µg/band	0.1 µg/band	0.1 µg/band	80	Our work

## Conclusion

In conclusion, HPTLC densitometric and smartphone-assisted HPTLC methods were successfully developed and validated for the simultaneous determination of VON and ASP in pharmaceutical formulations. The optimized chromatographic conditions yielded well-resolved, symmetric peaks, ensuring reliable quantification. The smartphone-assisted approach, which utilizes ImageJ analysis of plate images, proved to be an innovative, simple, and cost-effective alternative to conventional HPTLC densitometry, offering comparable accuracy and precision. The methods complied with ICH validation guidelines, confirming their suitability for routine analysis of VON and ASP in pure form and dosage formulations. Notably, the smartphone-assisted method—requiring only TLC plates and a UV lamp—enhances accessibility, minimizes dependence on sophisticated instrumentation, and highlights the potential of smart phone assisted HPTLC as a sustainable tool in pharmaceutical analysis.

## Supplementary Information

Below is the link to the electronic supplementary material.

Supplementary Material 1

## Data Availability

All data generated or analysed during this study are included in this published article [and its supplementary information files].

## References

[1] St, E., Onge, B. & Phillips Vonoprazan: a new potassium-competitive acid blocker. *J. Pharm. Technol.* **39**, 139–146 (2023). 37323765 10.1177/87551225231166531 PMC10268044

[2] Akazawa, Y., Fukuda, D. & Fukuda, Y. Vonoprazan-based therapy for Helicobacter pylori eradication: experience and clinical evidence, therap. *Adv. Gastroenterol.* **9**, 845–852 (2016). 10.1177/1756283X16668093 PMC5076777 27803739

[3] Alzaghal, N. M. & El-Mossalamy, E. S. H. El-sayed, method development and validation for Estimation of Vonoprazan by RP-HPLC method in bulk and tablets dosage form. *Egypt. J. Chem.* **67**, 145–159 (2024).

[4] Luo, Z. et al. Development of a stability–indicating HPLC method for simultaneous determination of ten related substances in Vonoprazan fumarate drug substance. *J. Pharm. Biomed. Anal.* **149**, 133–142 (2018). 29112902 10.1016/j.jpba.2017.11.011

[5] Barseem, A., Elshahawy, M. & Elagamy, S. H. Fluorimetric determination of Vonoprazan via quenching of nitrogen and sulfur co-doped carbon quantum dots: A rapid and sustainable analytical approach. *Luminescence* **39**, e4834 (2024). 39036968 10.1002/bio.4834

[6] Saraya, R. E., Hassan, Y. F., Eltukhi, W. E. & Salman, B. I. Ultra-sensitive fluorimetric method for the first Estimation of Vonoprazan in real human plasma and content uniformity test. *J. Fluoresc*. **32**, 1725–1732 (2022). 35670919 10.1007/s10895-022-02979-2 PMC9402479

[7] Mahgoub, H., Ragab, M. A. A., Tarek, S. & Maher, H. M. An eco-friendly liquid chromatographic analysis of the triple therapy protocol of amoxicillin, metronidazole and Vonoprazan for H. Pylori eradication: application to combined dosage forms and simulated gastric fluid. *BMC Chem.* **18**, 106 (2024). 38816886 10.1186/s13065-024-01210-6 PMC11138008

[8] Aboras, S. I., Ahmed, A. R., Belal, T. S. & Elbordiny, H. S. A tri-hued and sustainable assessment of HPLC for the concurrent determination of multi-purpose binary and ternary Vonoprazan combinations: application to combined dosage forms and simulated gastric juice. *Microchem J.* **213** 113882. (2025).

[9] Kontovas, S., Misailidis, N., Mustafa, A. & Petrides, D. Aspirin Production, (2023).

[10] Trapali, M. I. *Therapeutic Uses of Aspirin, In: Pain Manag* (Acute to Chronic Beyond, 2023).

[11] Panahi, H., Rahimi, A., Moniri, E., Izadi, A. & Parvin, M. HPTLC separation and quantitative analysis of aspirin, Salicylic acid, and sulfosalicylic acid, JPC-Journal planar chromatogr. *TLC* **23**, 137–140 (2010).

[12] Porwal, P. K., Ahmad, A., Chhajed, R. A. S. S. & Chatpalliwar, V. A. Liquid chromatographic method for simultaneous quantitation of clopidogrel, aspirin and Atorvastatin in rat plasma and its application to the Pharmacokinetic study. *J. Chromatogr. Sci.* **53**, 1155–1162 (2015). 25609600 10.1093/chromsci/bmu210

[13] Goyal, R. N., Bishnoi, S. & Agrawal, B. Electrochemical sensor for the simultaneous determination of caffeine and aspirin in human urine samples. *J. Electroanal. Chem.* **655**, 97–102 (2011).

[14] Ismail, A., Gamal, M. & Nasr, M. Optimization of analytical method for simultaneous determination of acetaminophen, caffeine, and aspirin in tablet dosage form. *Pharm. Chem. J.* **56**, 1682–1688 (2023).

[15] Murtaza, G. et al. Development of a UV-spectrophotometric method for the simultaneous determination of aspirin and Paracetamol in tablets. *Sci. Res. Essays*. **6**, 417–421 (2011).

[16] Mo\ct, A. C., Soponar, F., Medvedovici, A. & Sârbu, C. Simultaneous spectrophotometric determination of aspirin, paracetamol, caffeine, and chlorphenamine from pharmaceutical formulations using multivariate regression methods. *Anal. Lett.* **43**, 804–813 (2010).

[17] Abdelazim, A. H., Abdel-Fattah, A., Osman, A. O. E., Abdel-Kareem, R. F. & Ramzy, S. Spectrophotometric quantitative analysis of aspirin and Vonoprazan fumarate in recently approved fixed-dose combination tablets using ratio spectra manipulating tools. *J. AOAC Int.* **106**, 490–495 (2023). 36264114 10.1093/jaoacint/qsac128

[18] Moneim, M. M. A. & Hamdy, M. M. A. Chromatographic assay of recently approved co-formulation of Vonoprazan fumarate with low dose aspirin: AGREE, complex MoGAPI, and RGB 12-model assessments. *BMC Chem.* **18**, 230 (2024). 39548471 10.1186/s13065-024-01344-7 PMC11568669

[19] Schroeder, A. B. et al. The ImageJ ecosystem: Open-source software for image visualization, processing, and analysis. *Protein Sci.* **30**, 234–249 (2021). 33166005 10.1002/pro.3993 PMC7737784

[20] Moaaz, E. M., Abdel-Moety, E. M., Rezk, M. R. & Fayed, A. S. An eco-friendly smartphone based HPTLC method versus conventional densitometric one for determination of Naltrexone and bupropion. *BMC Chem.* **18**, 185 (2024). 39313836 10.1186/s13065-024-01285-1 PMC11421204

[21] Moaaz, E. M., Abdel-Moety, E. M., Rezk, M. R. & Fayed, A. S. Smartphone based TLC approach versus conventional densitometric measurement for the simultaneous determination of donepezil and memantine, content uniformity testing along with greenness and whiteness assessment. *Sustain. Chem. Pharm.* **42**, 101789 (2024).

[22] Ristivojević, P., Trifković, J., Vovk, I. & Milojković-Opsenica, D. Comparative study of different approaches for multivariate image analysis in HPTLC fingerprinting of natural products such as plant resin. *Talanta* **162**, 72–79 (2017). 27837887 10.1016/j.talanta.2016.10.023

[23] Kubheka, N. P. et al. ImageJ analysis for quantifying lead ion in environmental water using gold nanoparticles as a colorimetric probe. *J. Mol. Liq*. **420**, 126804 (2025).

[24] Chang, Y. C., Wu, K. H., Wang, J. C. & Huang, W. C. ImageJ and smartphone app chemistry analyzer using image-based colorimetric assay for quantitative detection of melamine. *Pure Appl. Chem.* **97**, 235–244 (2025).

[25] Elagamy, S. H., Adly, L., Abdel, M. A., Hamid & Image, J. Smartphone based colorimetric approach for quantitative determination of uric acid using *Sci. Rep.* **13** 21888. (2023). 38081872 10.1038/s41598-023-48962-0 PMC10713523

[26] Paper, W. Understanding the Latest Revisions to USP < 621 > method transfers and modernization of LC methods, (n.d.).

[27] Organization, W. H. *WHO Expert Committee on Specifications for Pharmaceutical Preparations: fifty-sixth Report* (World Health Organization, 2022).

[28] ICH, ICH Harmonised Guidance:Validation of Analytical Procedures Q2(R & Harmon, I. C. H. 2), *Tripart Guidel* **2** 1–34. https://database.ich.org/sites/default/files/Q1A%28R2%29 Guideline.pdf . (2022).

[29] Hammad, S. F., Hamid, M. A. A., Adly, L. & Elagamy, S. H. Comprehensive review of greenness, whiteness, and blueness assessments of analytical methods. *Green. Anal. Chem.* **12** 100209. (2025).

[30] Gałuszka, A., Migaszewski, Z. M., Konieczka, P. & Namieśnik, J. Analytical eco-scale for assessing the greenness of analytical procedures. *TrAC Trends Anal. Chem.* **37**, 61–72 (2012).

[31] Yin, L. et al. Green analytical chemistry metrics for evaluating the greenness of analytical procedures. *J. Pharm. Anal.* **14** 101013. (2024). 10.1016/j.jpha.2024.101013 PMC11697060 39759968

[32] Manousi, N., Wojnowski, W., Płotka-Wasylka, J. & Samanidou, V. Blue applicability grade index (BAGI) and software: a new tool for the evaluation of method practicality. *Green. Chem.* **25**, 7598–7604. 10.1039/d3gc02347h (2023).

[33] Nowak, P., Wietecha-Posłuszny, R. & Pawliszyn, J. White analytical chemistry: an approach to reconcile the principles of green analytical chemistry and functionality. *TrAC Trends Anal. Chem.* **138**, 116223 (2021).

[34] Pingili, D., Awasthi, A. & Varshney, M. White analytical chemistry: a review of current developments. *J. Anal. Chem.* **80**, 959–968. 10.1134/S1061934825700327 (2025).

[35] Hussain, C. M., Hussain, C. G. & Keçili, R. White analytical chemistry approaches for analytical and bioanalytical techniques: applications and challenges. *TrAC Trends Anal. Chem.* **159**, 116905 (2023).

