# Supplementary material for: Smartphone-assisted HPTLC for simultaneous determination of vonoprazan fumarate and aspirin: a comparative study with HPTLC densitometry
Source: Sci Rep. 2025 Nov 24;15:41809. doi: 10.1038/s41598-025-26418-x (PMC12647887; doi:10.1038/s41598-025-26418-x)
Supplement: Supplementary file 1 — Supplementary Material 1 [file 41598_2025_26418_MOESM1_ESM.docx]

**Smartphone-Assisted HPTLC for Simultaneous Determination of Vonoprazan Fumarate and Aspirin: A Comparative Study with HPTLC Densitometry**


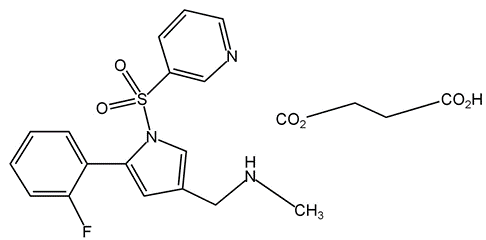


**Figure S1:** Chemical structure of vonaprazan fumarate.


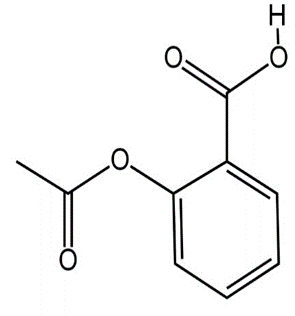


**Figure S2:** Chemical structure of aspirin


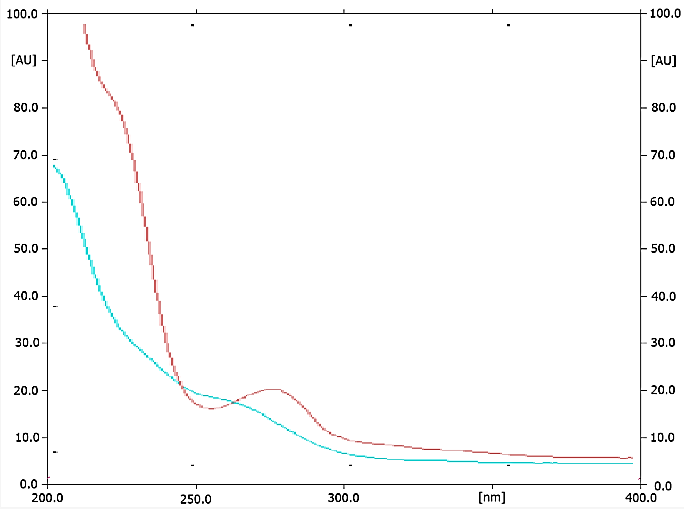


270 nm

Figure S3 : Densitometrically recorded absorption spectrum of VON ( )and ASP( .)


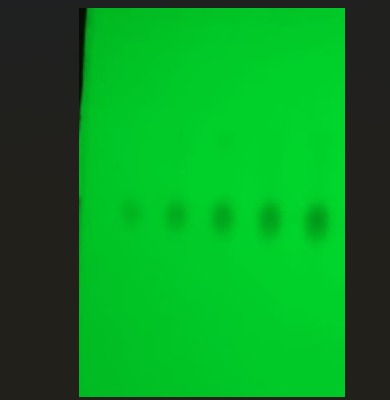
**
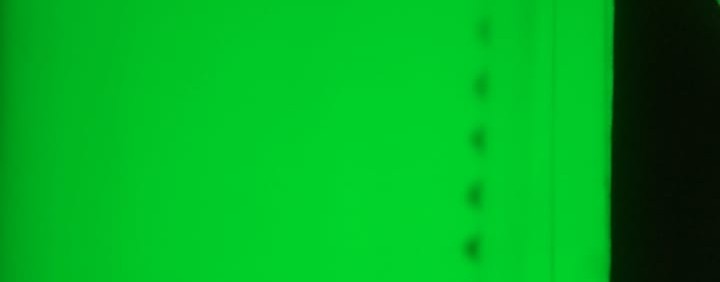
**

B

A

Figure S4. Original full-length TLC silica gel plates for (a) VON and (b) ASP corresponding to Figure 3.

**Table S1**. Evaluation of the accuracy for determination VON using HPTLC densitometric method and the ImageJ-based approach

| **Conc. taken** | **HPTLC** | | **HPTLC/IMAGE J** | |
| --- | --- | --- | --- | --- |
|  | **Mean* Conc.** | **Mean* % Recovery** | **Mean* Conc.** | **Mean* %Recovery** |
| **1.0** | 1.021 | 99.34 | 1.02 | 100.83 |
| **8.0** | 8.02 | 100.28 | 8.07 | 100.99 |
| **10** | 9.98 | 99.86 | 9.96 | 99.68 |

***Average of three determinations**

**Table S2**. Evaluation of the accuracy for determination ASP using HPTLC densitometric method and the ImageJ-based approach

| **Conc. taken** | **HPTLC** | | **HPTLC/IMAGE J** | |
| --- | --- | --- | --- | --- |
|  | **Mean* Conc.** | **Mean* % Recovery** | **Mean* Conc.** | **Mean* % Recovery** |
| **5.0** | 5.00345 | 100.03 | 4.98 | 99.61 |
| **15** | 15.10671 | 100.17 | 14.98 | 99.96 |
| **25** | 24.816 | 99.27 | 25.27 | 101.06 |

***Average of three determinations**

**Table S3:** Evaluation of the precision for determination VON using HPTLC densitometric method and the ImageJ-based approach

| **Conc. taken** | **HPTLC** | | | | **HPTLC/IMAGE J** | | | |
| --- | --- | --- | --- | --- | --- | --- | --- | --- |
|  | **Intraday** | | **Interday** | | **Intraday** | | **Interday** | |
|  | **Mean* % Recovery** | **SD** | **Mean* % Recovery** | **SD** | **Mean* % Recovery** | **SD** | **Mean* % Recovery** | **SD** |
| **2.0** | 99.36 | 0.83 | 100.12 | 1.00 | 101 | 1.32 | 101.03 | 1.30 |
| **4.0** | 99.46 | 0.56 | 101.02 | 0.65 | 100.04 | 0.92 | 101.3 | 1.01 |
| **7.0** | 100.46 | 0.90 | 101.18 | 0.84 | 100.17 | 1.77 | 100.69 | 1.34 |

***Average of three determinations**

**Table S4:** Evaluation of the precision for determination ASP using HPTLC densitometric method and the ImageJ-based approach

| **Conc. taken** | **HPTLC** | | | | **HPTLC/Image J** | | | |
| --- | --- | --- | --- | --- | --- | --- | --- | --- |
|  | **Intraday** | | **Interday** | | **Intraday** | | **Interday** | |
|  | **Mean* % Recovery** | **SD** | **Mean* % Recovery** | **SD** | **Mean* % Recovery** | **SD** | **Mean* % Recovery** | **SD** |
| **5.0** | 99.336 | 0.61 | 100.43 | 0.77 | 100.86 | 1.10 | 100.45 | 0.88 |
| **10** | 101.30 | 0.65 | 100.70 | 0.42 | 100.73 | 1.13 | 99.46 | 0.90 |
| **35** | 99.59 | 0.98 | 99.40 | 0.56 | 99.2 | 1.36 | 100.40 | 0.95 |

***Average of three determinations**

**Table S4. Calculation of Analytical Eco-Scale scores for the developed methods in comparison with reported HPTLC and HPLC method.**

| Parameters | Penalty Points (PPs) | | | |
| --- | --- | --- | --- | --- |
|  | The developed HPTLC densitometric Method | The developed HPTLC /IMAGE J | Reported HPTLC method [16] | Reported HPLC Method [16] |
| Reagents |  |  |  |  |
| Type (No. of pictogram and signal word ) | Methanol (3*2)  Methylene chloride (2*2)  Glacial acetic acid (2*2) | | Ethanol (2*2)  Ethyl acetate(2*2)  Ammonia(4*2) | Acetonitrile (2*2) |
| Amount  (hazard* amount) | <10 ml  (14*1=14) | | <10 ml  (16*1=16) | 10 to 100 ml  (2*2*2=8)  Total of 8 |
| Instrument |  |  |  |  |
| Energy consumption | <1.5 kW/h (1) | NO energy consumption (0) | <1.5 kW/h (1) | <1.5 kW/h (1) |
| Emission of vapors or gasses | 0 | 0 | 0 | 0 |
| Waste |  |  |  |  |
| Waste generated | 1 to 10 mL (3) | 1 to 10 mL (3) | 1 to 10 mL (3) | >10 mL (5) |
| Waste treatment | No treatment (3) | No treatment (3) | No treatment (3) | No treatment (3) |
| Total PPs | 21 | 20 | 23 | 17 |
| Score (100-PPs) | 79 | 80 | 77 | 83 |

**Appendix A**

Steps for using Image J for measurement of peak area measurement

1-Select File → Open from the menu bar to open a stored image file.

2-Select rectangular selection from the tool bar to draw a rectangle around the bands

3-Select Analyze→ Gels→Select first lane

3-Select Analyze→ Gels→ plot lanes

5- Select line from the tool bar to draw a baseline for each peak

6- Select wand from the tool bar to highlight the peak and measure its corresponding peak area.
